# Author Correction: Spiraling pathways of global deep waters to the surface of the Southern Ocean

**DOI:** 10.1038/s41467-017-02105-y

**Published:** 2018-01-15

**Authors:** Veronica Tamsitt, Henri F. Drake, Adele K. Morrison, Lynne D. Talley, Carolina O. Dufour, Alison R. Gray, Stephen M. Griffies, Matthew R. Mazloff, Jorge L. Sarmiento, Jinbo Wang, Wilbert Weijer

**Affiliations:** 1 Scripps Institution of Oceanography, La Jolla, CA 92093 USA; 2 Princeton University, Princeton, NJ 08544 USA; 3 Geophysical Fluid Dynamics Laboratory, Princeton, NJ 08540 USA; 4 Jet Propulsion Laboratory, California Institute of Technology, Pasadena, CA 91109 USA; 5 Los Alamos National Laboratory, Los Alamos, NM 87545 USA; 6 Present Address: Massachusetts Institute of Technology and Woods Hole Oceanographic Institution Joint Program in Oceanography, Cambridge, MA USA; 7 Australian National University, Canberra, ACT 2602 Australia

*Nature Communications* **8**:172 10.1038/s41467-017-00197-0; Article published online: 2 August 2017

The original version of this Article contained errors in Fig. 6. In panel a, the grey highlights obscured the curves for CESM, CM2.6 and SOSE, and the labels indicating SWIR, KP, MR, PAR, and DP were inadvertently omitted. These have now been corrected in both the PDF and HTML versions of the Article.

