# Supplementary material for: Spiraling pathways of global deep waters to the surface of the Southern Ocean
Source: Nat Commun. 2017 Aug 2;8:172. doi: 10.1038/s41467-017-00197-0 (PMC5541074; doi:10.1038/s41467-017-00197-0)
Supplement: Supplementary file 1 — Supplementary Information [file 41467_2017_197_MOESM1_ESM.pdf]

File Name: Supplementary Information
Description: Supplementary Figures, Supplementary Table, Supplementary Notes and Supplementary References

File Name: Supplementary Movie 1
Description: Deep water upwelling pathways from the Atlantic Ocean. Cumulative modeled (CM2.6) particle pathways for the first 50 years of the experiment from the Atlantic Ocean, with particles released in the depth range 1000-3500 m along 30°S. At each timestep, colored boxes mark 1° latitude x 1° longitude x 100 m depth grid boxes visited by more than 2.25 % of the total upwelling particle-transport from release at 30°S to the current timestep. Boxes are colored by depth.

File Name: Supplementary Movie 2
Description: Deep water upwelling pathways from the Indian Ocean. Cumulative modeled (CM2.6) particle pathways for the first 50 years of the experiment from the Indian Ocean, with particles released in the depth range 1000-3500 m along 30°S. At each timestep, colored boxes mark 1° latitude x 1° longitude x 100 m depth grid boxes visited by more than 2.25 % of the total upwelling particle-transport from release at 30°S to the current timestep. Boxes are colored by depth.

File Name: Supplementary Movie 3
Description: Deep water upwelling pathways from the Pacific Ocean. Cumulative modeled (CM2.6) particle pathways for the first 50 years of the experiment from the Pacific Ocean, with particles released in the depth range 1000-3500 m along 30°S. At each timestep, colored boxes mark 1° latitude x 1° longitude x 100 m depth grid boxes visited by more than 2.25 % of the total upwelling particle-transport from release at 30°S to the current timestep. Boxes are colored by depth.

File Name: Peer Review File
Description:

## Supplementary Information

### Supplementary Note 1

A motivation for this study is the set of maps in the WOCE Hydrographic Programme (WHP) atlas Volume 1: Southern Ocean (Orsi & Whitworth, 2005), with properties displayed along neutral density surfaces characterizing the Indian and Pacific Deep Waters (27.84 kg m$^{-3}$) and the North Atlantic Deep Water (28.05 kg m$^{-3}$). These maps were constructed by gridding hydrographic data from the National Oceanographic Data Center (NODC) and the WHP data collected in the 1990s. Fig. 1 and Supplementary Fig. 1 show properties along the latter neutral density surface, along with the four major ACC fronts from one particular source (Orsi et al., 1995), bathymetry (single contour of 3000 m is superimposed), and the latitude band of Drake Passage, across which the deep waters must move in order to upwell to the sea surface. The gridded potential temperature shown in Supplementary Fig. 1b was replotted on the gridded depth from Supplementary Fig. 1a to produce the 3-D Fig. 1a, selecting only the region where gridded potential temperature is greater than 1.6 °C. From the full map, it is clear that water well above freezing reaches the Antarctic continent in the southeastern Pacific sector, as reviewed in the main text. The oxygen and nitrate maps show the inflow of low oxygen, high nutrient waters from the Pacific and Indian Oceans, and their spiral around Antarctica, rising to the sea surface as tracked using depth in Supplementary Fig. 1a.

Careful attention to the pathway of the warmest water and most extreme oxygen and nitrate in Supplementary Fig. 1 suggests that the cores of most extreme properties shift southward across the ACC fronts over or downstream of the major topographic obstacles, particularly noticeable at the Mid-Atlantic Ridge, Southwest Indian Ridge and the Pacific-Antarctic Ridge. However, there is not enough resolution in these fields to definitively show details of shifts across fronts, and upwelling of the core of properties associated with these obstacles is difficult to extract from the hydrographic observations. The numerical model analyses clearly show both of these. These maps also do not provide transports or time scales for the upwelling water, nor do they clearly show where the properties preferentially reach the sea surface. And finally they cannot show diapycnal transformation along the pathway of particles in the southeast spiral. The numerical models are used to explore all of these important aspects of the upwelling pathways and rates.

### Supplementary Note 2

3-dimensional upwelling pathways in CM2.6 from the Indian and Pacific basins are similar to the Atlantic, as they show narrow boundary pathways connecting 30°S to the ACC (Supplementary Fig. 2 and Supplementary Videos 1-3). Figure 1b and Supplementary Figure 2 show the cumulative pathways of deep water after 200 years of the particle-release experiment, so any information about the timescales of these pathways is not shown. To look at how the pathways evolve over time from the initial release of particles at 30°S, Supplementary Videos 1-3 show the 3-dimensional particle pathways at monthly snapshots from the time of release up to 50 years.

### Supplementary Note 3

Unresolved sub-grid scale physics and dynamics and temporal averaging of the model velocities have an impact on particle trajectories. Thus we performed multiple sensitivity analyses using SOSE to assess the

impact of varying model averaging timescales and Lagrangian methods on our results. First, the impact of adjusting the temporal averaging of model velocity data is compared for daily averaged, 5-day averaged and 30-day averaged velocities (Supplementary Fig. 5). SOSE velocities were saved as daily averaged output, but due to limited computer resources CM2.6 velocity output is only available as 5-day averages and CESM output is only available as 30-day averages. The maximum storage interval for accurate Lagrangian particle tracking depends on several factors related to dominant scales of length, velocity, model grid size and time. Experiments in the global 1/10° OFES model (the same resolution as CM2.6 and CESM used here) showed that connectivity transports and transit times (bulk measure of the flow) were relatively insensitive to time averaging on timescales of 3-days to 30-days (Qin et al., 2014). Here we compare the impact of time-averaging velocities on our particular results, and we use SOSE because it has sufficiently high temporal resolution output available to test this (Supplementary Fig. 3), while CESM has only 30 day averaged output. We find that pathways are insensitive to averaging velocities on 5-day timescales compared to daily timescales north and within the ACC, but south of the ACC particle-transport is slightly higher in the 5-day averaged experiment than the daily averaged experiment (Supplementary Fig. 3b,d). The 30-day averaged experiment is also qualitatively similar to the daily and 5-day averaged experiment, but with somewhat less particle-transport in the ACC and more particle-transport south of the ACC than the experiment using daily averages (Supplementary Fig. 3c,e). It is important to note that only single-particle statistics are used in this analysis, which are expected to be mainly affected by the most energetic parts of the flow and thus are less sensitive to sampling frequency than other Lagrangian statistics. This could explain why the CESM results are similar to CM2.6 and SOSE even though the mesoscale is not well sampled in CESM. Further work is needed to fully interpret the differences in the experiments with different timescale averages, but the qualitative agreement of the upwelling pathways in SOSE with velocity averaging timescales up to 30-days suggest that our analysis of 30-day averaged velocities in CESM is justified.

Second, for some applications it is useful to add stochastic noise to trajectories with the aim of parameterizing diffusion by unresolved eddy motions that are absent in the explicitly resolved eddy field. Because the models used here are eddy-resolving or eddy-permitting, we have chosen not to include stochastic noise in the trajectory motions. To test whether the inclusion of stochastic noise significantly impacts our results, we repeated the experiment in SOSE with the addition of a random walk scheme. An additional displacement is added to particles at each time step by implementing the random number generator algorithm described in Kinderman & Monahan (1977) with zero mean and unit variance and a horizontal diffusivity of 25 $m^2$ $s^{-1}$ and a vertical diffusivity of $1x10^{-5}$ $m^2$ $s^{-1}$. We show in Supplementary Figure 4 that the resulting pathways do not change significantly with the inclusion of diffusion. With diffusion, there is slightly less particle-transport that travels close to the Antarctic continent and slightly less particle-transport following the Agulhas Current southward from 30°S, and a very slight increase in particle-transport within the ACC. There are several limitations to this comparison that are important to note. First, the stochastic noise we added to the trajectories is unbiased (positive and negative displacements are equally probable), which we know is not true in reality. Secondly, there are important sub-mesoscale advection and mixing processes not resolved in the models that are not parameterized. We acknowledge these limitations, and while the comparison in Supplementary Figure 4 is a first step toward determining the impact of mixing on particle trajectories, further work is needed to represent mixing processes more realistically.

Finally, sufficiently large numbers of particle trajectories are important to accurately represent the

volume transport and provide robust statistics. We test the sensitivity of the particle pathways to the number of particles released by halving the number of particle trajectories used in the pathway analysis in SOSE (Supplementary Fig. 5). The comparison shows that the upwelling pathways are insensitive to halving the number of particles, indicating that we have released sufficient numbers of particles to capture the spatial structure of the upwelling pathways.

**Supplementary Note 4**

Upwelling across the 1000 m depth surface in CESM and SOSE is similar to that in CM2.6 (Supplementary Figs. 6a and 7a). Note that because CESM output is 30-day averages, the eddy kinetic energy (contoured in blue in Fig. S6a) does not include transient mesoscale variability on timescales less than 30-days. However, the spatial patterns of high EKE in CESM are very similar to those in CM2.6, which includes variability between 5-days and 30-days.

The particle-transport crossing the 200 m depth surface varies more across models than the 1000 m depth crossings (Supplementary Figs. 6b and 7b) as it is influenced by mixed layer and upper ocean processes, which vary across the models.

| Model | | Median | Mean | Mode |
|---|---|---|---|---|
| CESM | Total | 70 | 79 | 41 |
| | Atlantic | 61 | 72 | 48 |
| | Indian | 68 | 78 | 50 |
| | Pacific | 58 | 68 | 41 |
| CM2.6 | Total | 62 | 72 | 28 |
| | Atlantic | 49 | 61 | 28 |
| | Indian | 68 | 78 | 48 |
| | Pacific | 47 | 60 | 22 |
| SOSE | Total | 92 | 96 | 81 |
| | Atlantic | 89 | 96 | 82 |
| | Indian | 76 | 82 | 29 |
| | Pacific | 109 | 111 | 93 |

Supplementary Table 1: Timescales of upwelling in each model and each ocean basin. The mode is determined by first smoothing the transit time distribution, then finding the maximum value in the distribution.

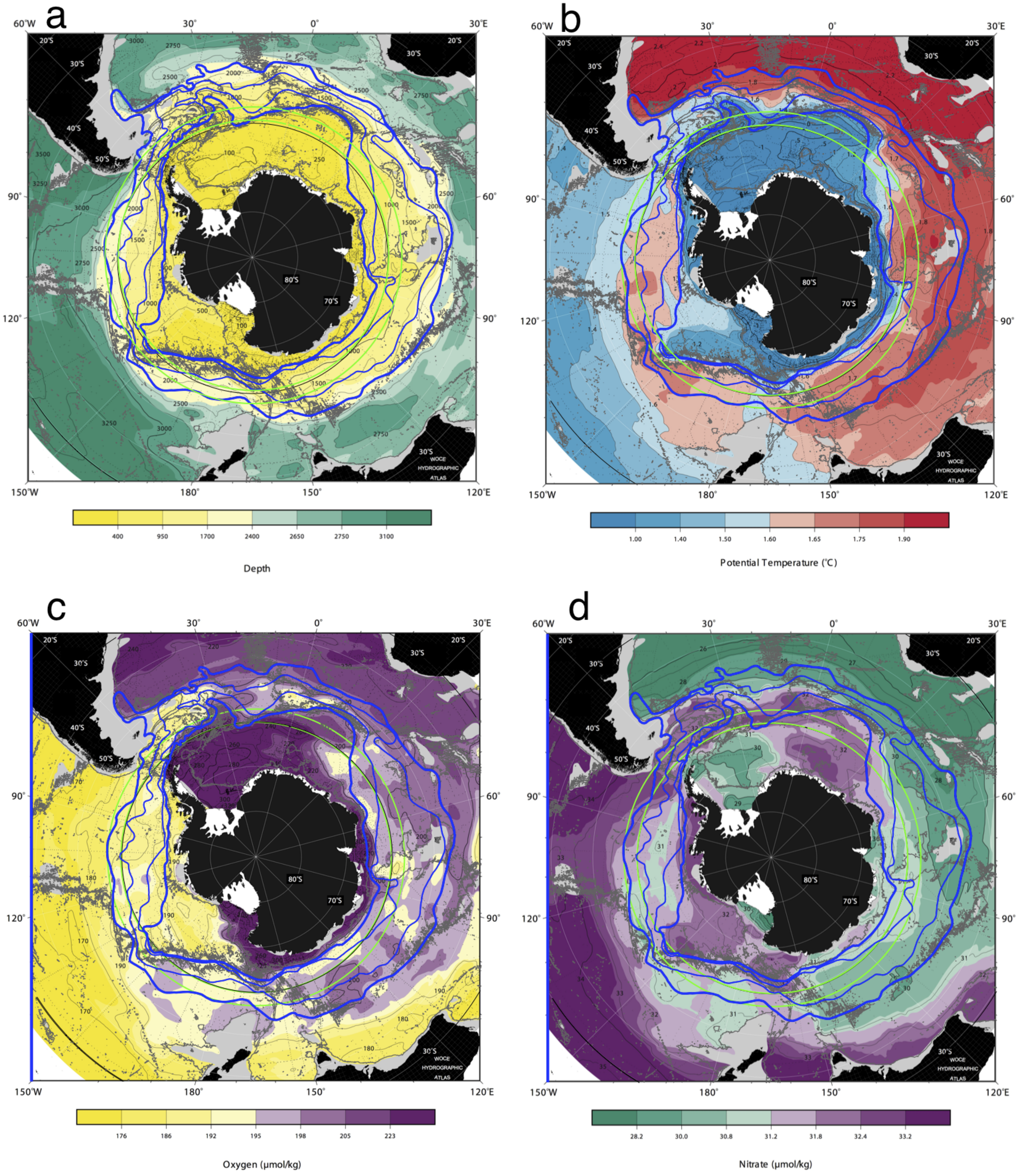


Supplementary Figure 1: Properties of the neutral density surface 28.05 kg m$^{-3}$, from the WOCE Hydrographic Programme Atlas Volume 1: Southern Ocean (Orsi & Whitworth, 2005):(a) Depth (m), (b) potential temperature (°C), (c) oxygen (umol/kg), (d) nitrate (umol/kg). Superimposed on the WHP maps are the 3000 m bathymetric contour (gray), ACC fronts (Orsi et al., 1995) (blue), and the latitude range of Drake Passage (green).

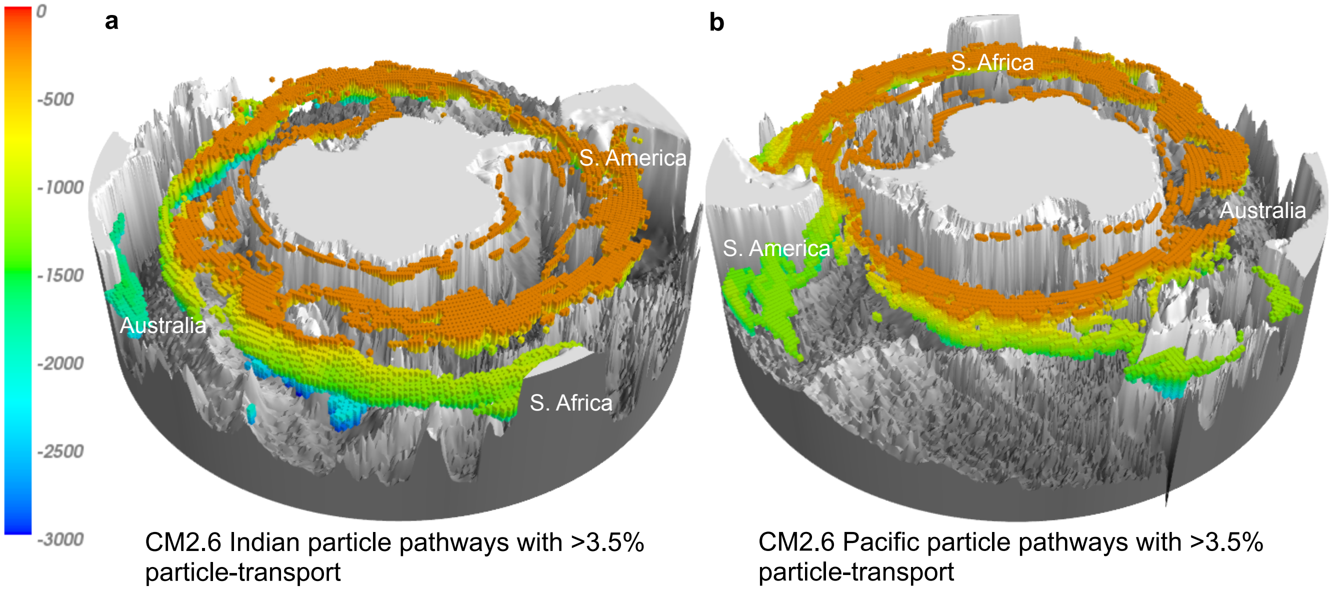


Supplementary Figure 2: Particle pathways in CM2.6 as in Figure 1b but for particles that originate in the (a) Indian Ocean and (b) Pacific Ocean. Particles leave the Indian Ocean along two pathways: in the Agulhas Return Current east of Africa and in a deeper tongue along the Australian south coast. Particles leave the Pacific Ocean along three pathways: from the Tasman Sea, from the New Zealand region, and between the East Pacific Rise and South America.

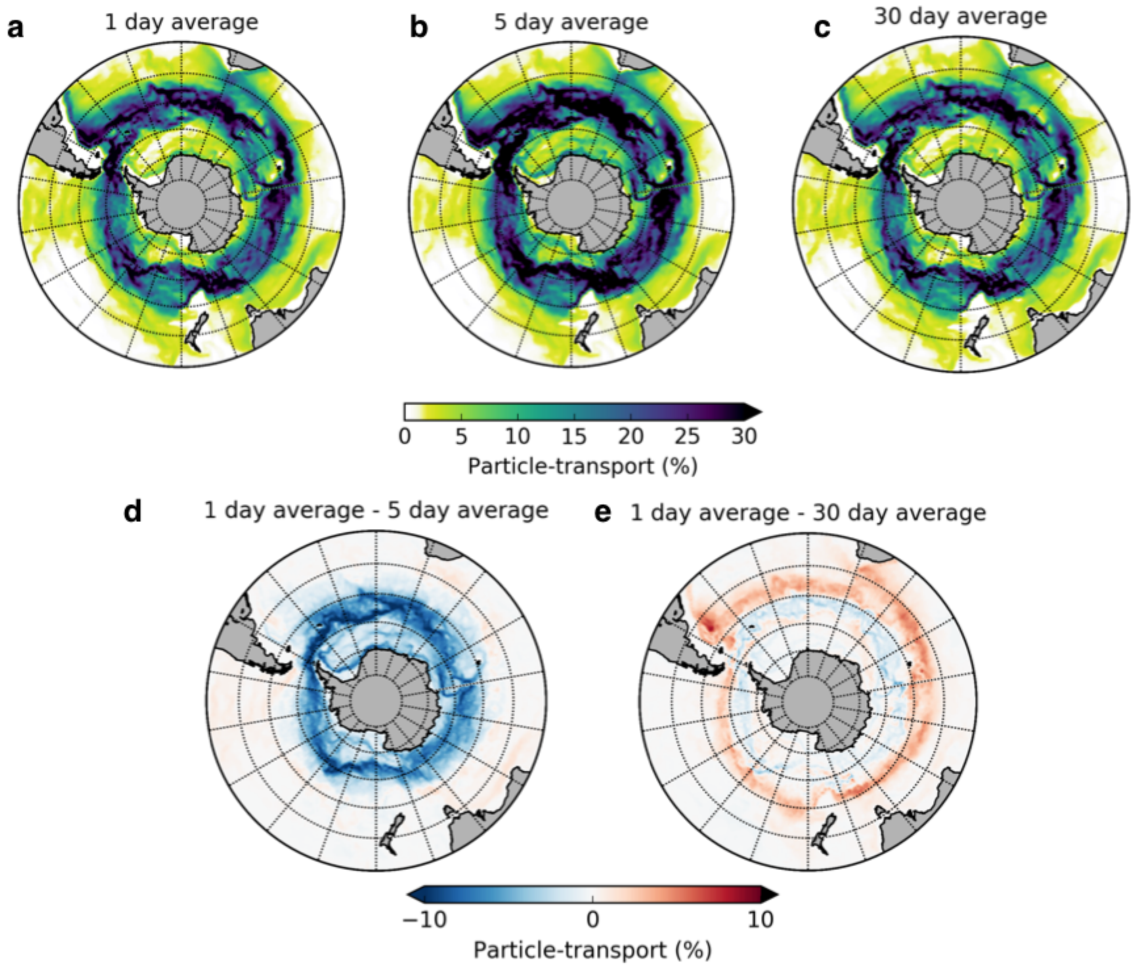


Supplementary Figure 3: Sensitivity of pathways in SOSE to velocity averaging. Percent of particle-transport visiting each 1° latitude x 1° longitude grid column from release at 30°S and before reaching the surface mixed layer for (a) SOSE daily averaged velocity output, (b) SOSE 5-day averaged velocity output, (c) SOSE 30-day averaged velocity output, (d) daily averaged velocities minus 5-day averaged velocities (a-b) and (e) daily averaged velocities minus 30-day averaged velocities (a-c). Note that the colorbar axis limits in panels (d) and (e) differ from (a), (b) and (c).

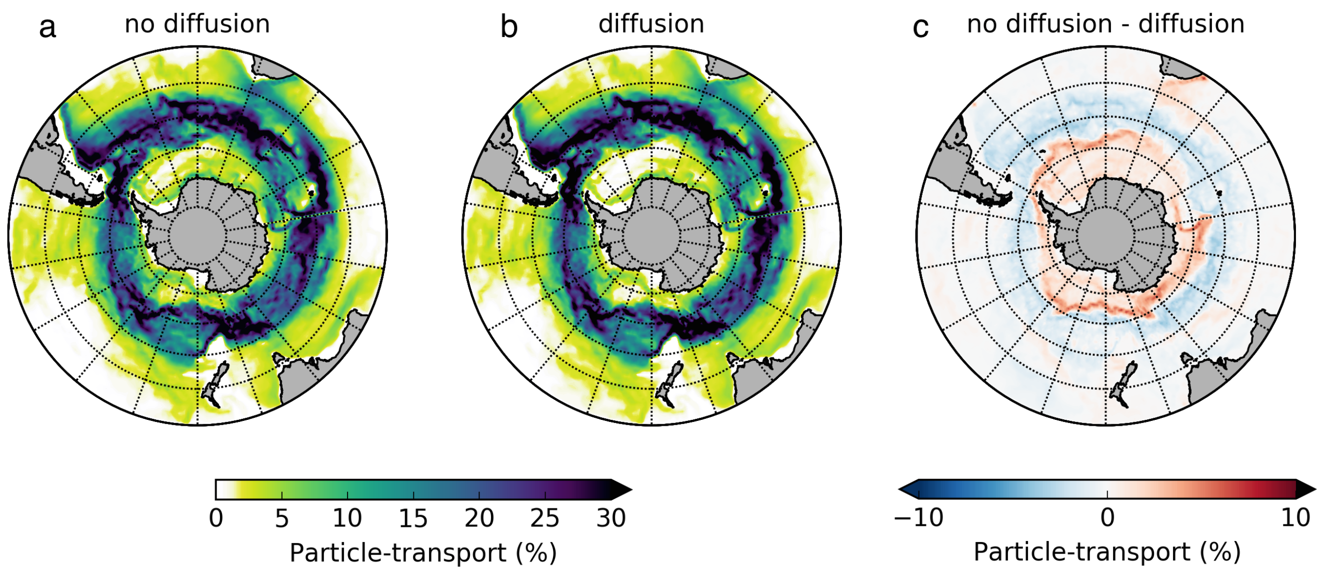


Supplementary Figure 4: Sensitivity of pathways in SOSE to inclusion of a stochastic noise component in the trajectory motion to represent sub-grid scale diffusion. Percent of particle-transport visiting each 1° latitude x 1° longitude grid column from release at 30°S and before reaching the surface mixed layer for (a) no diffusion, (b) with diffusion, and (c) no diffusion minus with diffusion (a-b).

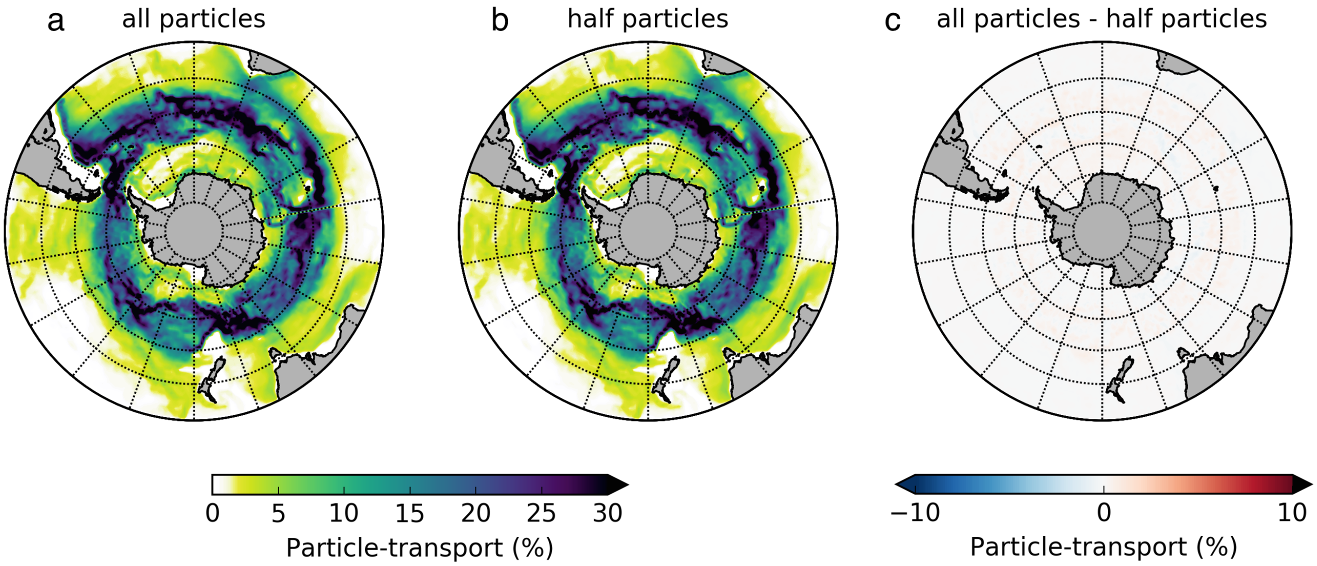


Supplementary Figure 5: Sensitivity of pathways in SOSE to halving the number of particles included in the pathway calculation. Percent of particle-transport visiting each 1° latitude x 1° longitude grid column from release at 30°S and before reaching the surface mixed layer for (a) all of the upwelled particle trajectories, (b) half of the total upwelled particle trajectories, and (c) all particle trajectories minus half of the particle trajectories (a-b).

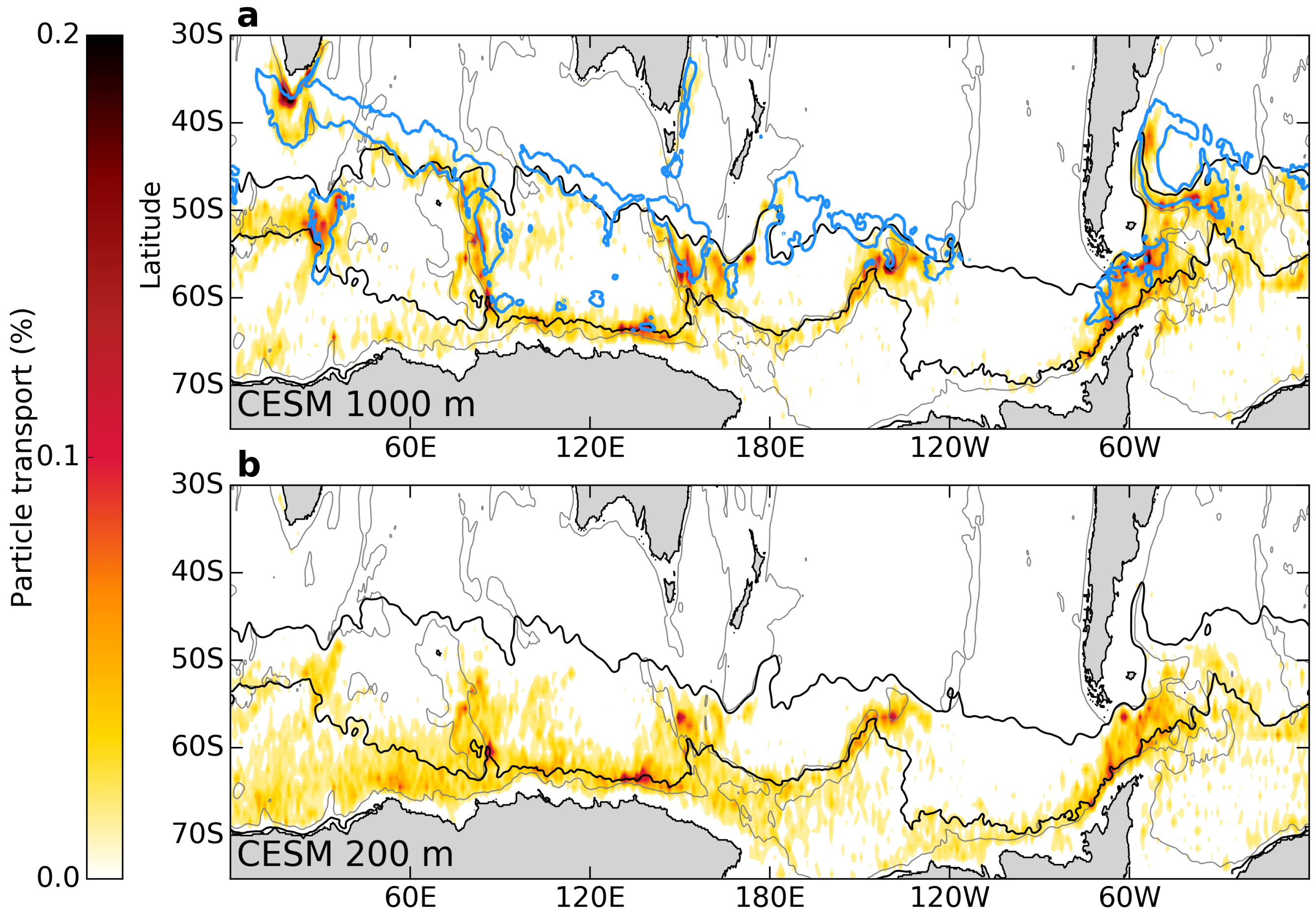


Supplementary Figure 6: Upwelling of particles across depth horizons. (a) Percent of particle-transport crossing 1000 m in each 1° latitude x 1° longitude grid box between release at 30° S and the mixed layer in CESM and (b) same as (a) for 200 m. In all panels, we select the location at which particles cross depth surfaces for the final time along their trajectories. Qualitatively similar results are obtained from selecting first crossing locations. Black contours are the outermost closed contours through Drake Passage of mean sea surface height in CESM, representing the path of the ACC. Blue contours in a) indicate regions where the eddy kinetic energy at 1000 m in CESM is higher than 75 $cm^2s^{-2}$.

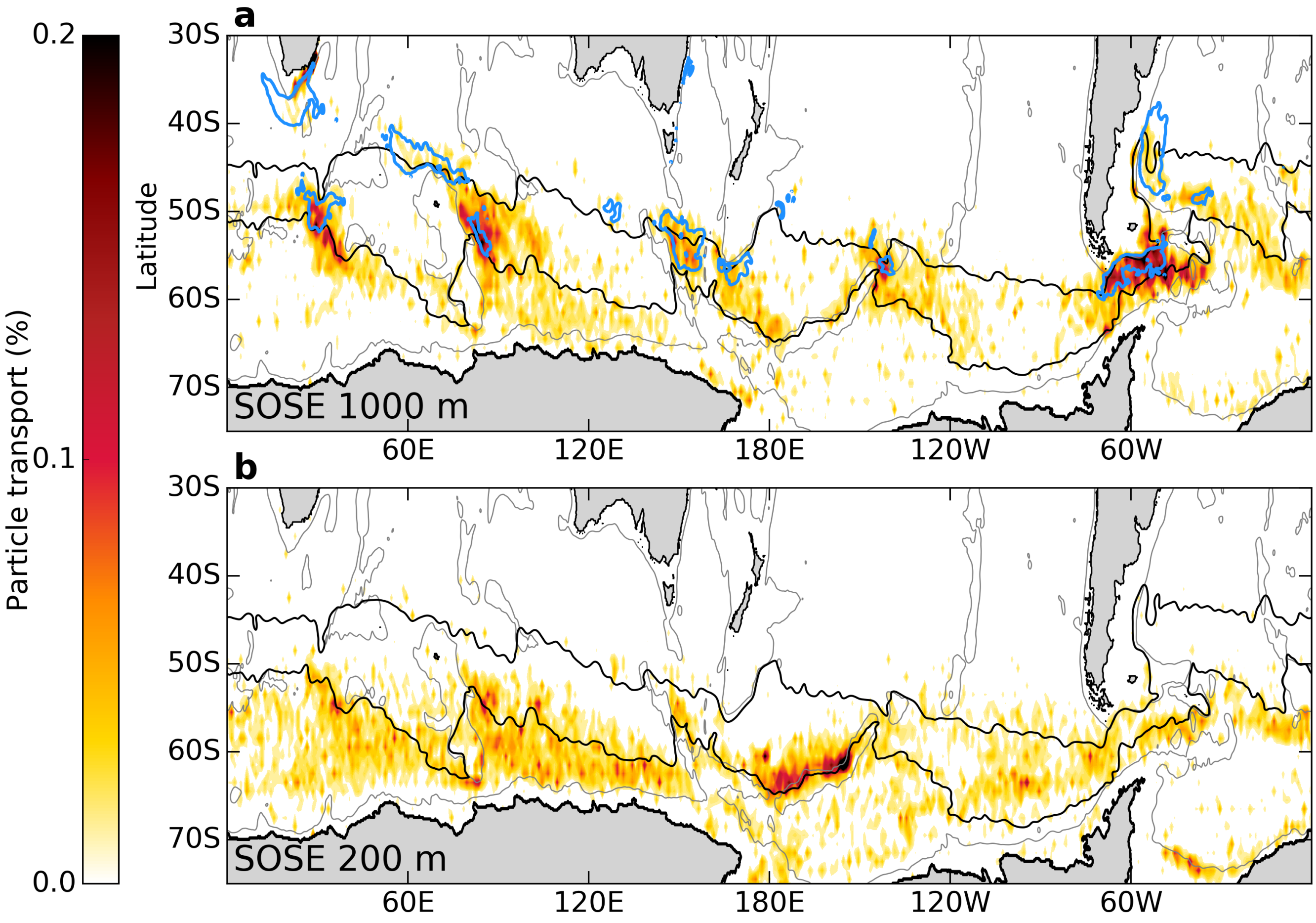


Supplementary Figure 7: Upwelling of particles across depth horizons. (a) Percent of particle-transport crossing 1000 m in each 1° latitude x 1° longitude grid box between release at 30° S and the mixed layer in SOSE and (b) same as a) for 200 m. In all panels, we select the location at which particles cross depth surfaces for the final time along their trajectories. Qualitatively similar results are obtained from selecting first crossing locations. Black contours are the outermost closed contours through Drake Passage of mean sea surface height in SOSE, representing the path of the ACC. Blue contours in (a) indicate regions where the eddy kinetic energy at 1000 m in SOSE is higher than 75 $cm^2 s^{-2}$.

## Supplementary References


Kinderman, A. & Monahan, J. (1977). Computer generation of random variables using the ratio of uniform deviates. *ACM Trans. Math. Softw.*, 3(3), 257–260.

Orsi, A. H. & Whitworth, T. (2005). *Hydrographic Atlas of the World Ocean Circulation Experiment (WOCE): Volume 1: Southern Ocean*. WOCE International Project Office.

Orsi, A. H., Whitworth, T., & Nowlin, W. D. (1995). On the meridional extent and fronts of the Antarctic Circumpolar Current. *Deep Sea Research Part I: Oceanographic Research Papers*, 42(5), 641–673.

Qin, X., van Sebille, E., & Gupta, A. S. (2014). Quantification of errors induced by temporal resolution on lagrangian particles in an eddy-resolving model. *Ocean Modelling*, 76, 20–30.
